# Role of circ_0012856 in modulating molecular pathways of diabetic peripheral neuropathy

**DOI:** 10.1002/ccs3.70019

**Published:** 2025-06-12

**Authors:** Ji Chen, Fan Zhang, Yangyuxi Chen, Yingqing Lu, Xinxin Liu, Yuanzhang Tang, Fengrui Yang

**Affiliations:** ^1^ Department of Endocrinology Hunan University of Medicine General Hospital Huaihua Hunan China; ^2^ Department of Anesthesiology Xiangya Hospital of Central South University Changsha Hunan Province China; ^3^ Yali High School International Department Changsha Hunan China; ^4^ Department of Anesthesiology Hunan University of Medicine General Hospital (Jishou University of Huaihua Hospital) Huaihua Hunan China; ^5^ Department of Pain Management The First Affiliated Hospital of Guangzhou Medical University Guangzhou Guangdong China

**Keywords:** autophagy, circ_0012856, diabetic peripheral neuropathy, EZH2, microglial polarization, MicroRNA‐124, STAT3

## Abstract

Diabetic peripheral neuropathy (DPN), a common complication of diabetes mellitus, involves complex molecular pathways and the ceRNA regulatory network. Integration of diabetes‐related circRNA datasets identified circ_0012856 as pivotal via least absolute shrinkage and selection operator and support vector machine recursive feature elimination algorithms. Functional enrichment analyses elucidated its involvement in DPN pathogenesis. In vitro studies showed circ_0012856 regulating EZH2 and STAT3 expressions, inhibiting autophagy, and promoting microglial M1 polarization. In vivo, experiments revealed silencing circ_0012856 alleviating DPN symptoms in diabetic mice. Overall, circ_0012856 acts as a miR‐124 sponge, affecting key pathways in DPN progression and providing potential therapeutic targets.

## Graphical abstract

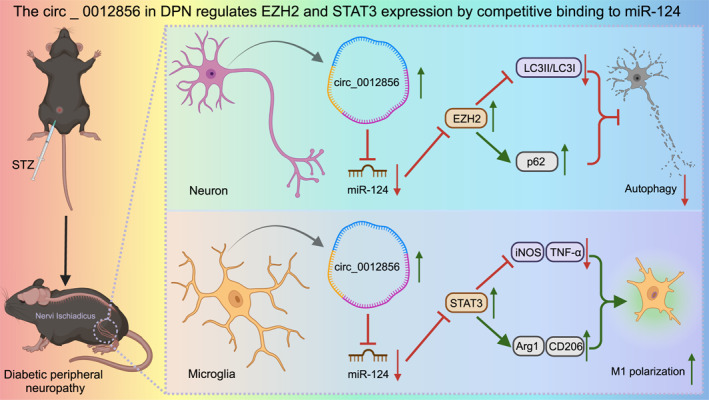

## 1 INTRODUCTION

Diabetic peripheral neuropathy (DPN) is one of the most common chronic complications in patients with diabetes (DM), with approximately 50% experiencing DPN symptoms during their lifetime.^1^ The clinical manifestations of DPN primarily include neuropathic pain, sensory abnormalities, muscle weakness, and reduced or absent deep tendon reflexes.^2^, ^3^ As the disease progresses, patients may develop mobility impairments and disabilities, reducing their quality of life and potentially leading to severe complications such as foot ulcers and amputations.^4^, ^5^ Currently, the pathogenesis of DPN is not fully understood, and existing treatments are limited to symptom management, with no effective etiological therapies available.^6^ Therefore, further investigation into the pathological mechanisms of DPN to identify new molecular targets and therapeutic strategies is of great clinical significance.

In recent years, a growing body of research has demonstrated that noncoding RNAs (ncRNAs) play a crucial role in the onset and progression of DPN. Notably, the competitive endogenous RNA (ceRNA) regulatory network involving circular RNAs (circRNAs), microRNAs (miRNAs), and messenger RNAs (mRNAs) has been shown to exert significant regulatory functions in various neurological disorders.^7^, ^8^ As ceRNAs, circRNAs regulate downstream gene expression by sponging specific miRNAs, thereby influencing cellular functions and pathological processes.^9^ Studies have already shown that several circRNAs exhibit abnormal expression in diabetic models and are closely associated with DM‐related metabolic disorders, inflammatory responses, and neural damage. However, research on the specific mechanisms by which circRNAs influence DPN progression through the ceRNA pathway remains relatively limited, warranting further investigation.

Research on circRNAs in neurological diseases has garnered increasing attention due to their stable circular structure, which grants them a longer half‐life and extensive biological activity within cells.^10^ In DPN, circRNAs may regulate downstream mRNA expression by competitively binding to miRNAs, thereby influencing the functions of neurons and glial cells.^11^, ^12^ Studies have identified circ_0012856 as a circRNA closely associated with DM, though its specific role and molecular mechanism in DPN remain unclear. microRNA‐124 (miR‐124), a highly conserved miRNA in the nervous system, regulates various neuronal activities and inflammatory responses, and its role in DPN has also gained considerable attention.^13^ Therefore, exploring the interaction between circ_0012856 and miR‐124, as well as the potential role of this mechanism in DPN, holds significant research value.

EZH2 and STAT3 are two key signaling molecules in the molecular mechanisms regulating neural cell functions. EZH2, the catalytic subunit of the histone methyltransferase complex, has been shown to play a critical role in neuronal autophagy, with EZH2 inhibition promoting autophagy activation.^14^, ^15^ Additionally, STAT3, a signal transducer and activator of transcription, is widely involved in cell proliferation, differentiation, and inflammatory responses and plays a particularly important role in the polarization of microglia.^16^, ^17^, ^18^ Microglial polarization is categorized into M1 and M2 types, with M1 polarization closely associated with enhanced inflammation and neuronal damage.^19^, ^20^, ^21^ Therefore, EZH2 and STAT3 may represent critical nodes in the pathological progression of DPN by regulating autophagy and inflammatory responses.

Based on the background above, this study aims to systematically investigate the specific role of circ_0012856 in the pathogenesis of DPN, with a particular focus on its mechanism of regulating EZH2 and STAT3 expression by sponging miR‐124. Through both in vivo and in vitro experiments, we validate the regulatory effects circ_0012856 on microglia's neuronal autophagy and M1 polarization and assess its impact on DPN progression. We hope this study will elucidate a novel molecular mechanism of circ_0012856 in DPN, providing new theoretical insights and potential targets for molecular diagnosis and targeted therapy of the condition. This research not only enhances the understanding of DPN pathogenesis but also lays the groundwork for developing novel therapeutic strategies with the potential to improve patient outcomes and quality of life.

## 2 MATERIALS AND METHODS

### 2.1 Data download and differential analysis

CircRNA‐related datasets GSE133225 and GSE231923 were downloaded from the GEO database (https://www.ncbi.nlm.nih.gov/geo/). GSE133225 includes peripheral blood mononuclear cell (PBMC) samples from 4 DM patients and 4 healthy individuals, whereas GSE231923 contains plasma samples from 6 DM patients and 6 healthy individuals. The GeneCards database (http://www.genecards.org/) was used to identify potential targets related to DPN. The “sva” R package (v3.5.0.0) was applied to remove batch effects between datasets, and differential expression analysis was performed using the “limma” R package (v3.58.1), with a threshold of *p* < 0.01. The results were visualized using volcano and heat maps.

### 2.2 Machine learning algorithms

Biomarkers were selected using a combination of least absolute shrinkage and selection operator (LASSO) logistic regression and support vector machine recursive feature elimination (SVM‐RFE) algorithms. The LASSO analysis used the R package “glmnet” (v4.1‐8) with the alpha parameter set to 1. For the SVM‐RFE analysis, the R package “e1071” (v1.7‐14) was used, with the number of top‐ranked features set to 50 and 10‐fold cross‐validation applied for model evaluation.

### 2.3 ROC analysis

Receiver operating characteristic (ROC) curves were plotted using the R package “pROC” (v1.18.5), and the area under the curve (AUC) was calculated to evaluate the genes' diagnostic potential.

### 2.4 Prediction of circRNA‐miRNA and miRNA‐mRNA interactions

Potential interactions between circRNAs and miRNAs were predicted using the circAtlas, miRDB, and circBank databases (http://www.circbank.cn/). The target genes of miRNAs were identified using the TargetScan, miRDB, and RNAInter databases.

### 2.5 GO/KEGG pathway enrichment analysis and PPI network construction

Gene ontology (GO) and Kyoto encyclopedia of genes and genomes (KEGG) pathway enrichment analyses were performed using the “clusterProfiler” R package (v4.10.1). The analysis identified enriched biological processes (BP), cellular components (CC), molecular functions (MF), and KEGG pathways. A threshold of *p* < 0.05 was considered statistically significant. The protein–protein interaction (PPI) network was constructed using the STRING database (https://www.string‐db.org/).

### 2.6 Transcription factor prediction and correlation analysis

The JASPAR database predicted transcription factor binding sites on gene promoters. Gene correlation analysis in brain and spinal cord samples was conducted using the Pearson method via the GEPIA2 database (http://gepia2.cancer‐pku.cn/#index).

### 2.7 Cell culture and grouping

Mouse microglial cells (BV2 cell line, SNL‐155, purchased from Sunncell, China) and mouse neuronal HT22 cells (SNL‐202, purchased from Sunncell, China) were cultured in DMEM (11965092, Gibco) supplemented with 10% fetal bovine serum (FBS, 12483020, Gibco). The culture medium was completely replaced every 3–4 days, and the cells were maintained under conditions of 37°C and 5% CO_2_.^22^, ^23^

Lentiviral vectors were used to construct EZH2 and STAT3 overexpression, circ_0012856 silencing, control groups, and miR‐124 inhibitor and NC inhibitor. The plasmids and lentiviral packaging services were provided by Sangon Biotech (Shanghai, China). Briefly, plasmids containing the target gene sequences were co‐transfected with helper plasmids into 293T cells (CRL‐3216, ATCC, USA). After validation, amplification, and purification, packaged lentiviruses were obtained. For lentiviral transfection of HT22 or BV2 cells, 5 × 10^5^ cells/well were seeded in 6‐well plates. When the cells reached 50%–70% confluence, they were infected with DMEM containing the appropriate amount of packaged lentivirus (multiplicity of infection, MOI = 10, with a working titer of approximately 5 × 10^6^ TU/mL) and polybrene (5 μg/mL) (TR‐1003, Merck, USA). After 4 h, an equal volume of fresh medium was added to dilute the polybrene. The medium was replaced after 24 h, and 48 h postinfection, the transfection efficiency was evaluated via fluorescence reporter gene expression. Stable transfected cell lines were selected using puromycin (5 μg/mL) (A1113803, Thermo Fisher).

To simulate a high‐glucose environment in the cell culture model, we first compared the effects of different glucose concentrations (25 mM, 30 mM, and 35 mM) on cellular pathology. Based on preliminary experiments (data not shown) and existing literature,^24^ we selected 30 mM glucose (15023021, Gibco) as the optimal concentration for simulating diabetic conditions in vitro.^24^

HT22 cells cultured under high‐glucose conditions were divided into the following groups: sh‐NC group (lentiviral infection with sh‐NC); sh‐circ_0012856 group (lentiviral infection with sh‐circ_0012856); sh‐circ_0012856 + NC inhibitor group (lentiviral infection with sh‐circ_0012856 and NC inhibitor); sh‐circ_0012856 + miR‐124 inhibitor group (lentiviral infection with sh‐circ_0012856 and miR‐124 inhibitor); sh‐NC + oe‐NC group (lentiviral infection with sh‐NC and oe‐NC); sh‐circ_0012856 + oe‐NC group (lentiviral infection with sh‐circ_0012856 and oe‐NC); and sh‐circ_0012856 + oe‐EZH2 group (lentiviral infection with sh‐circ_0012856 and oe‐EZH2).

BV2 cells cultured under high‐glucose conditions were divided into the following groups: sh‐NC group (lentiviral infection with sh‐NC); sh‐circ_0012856 group (lentiviral infection with sh‐circ_0012856); sh‐circ_0012856 + NC inhibitor group (lentiviral infection with sh‐circ_0012856 and NC inhibitor); sh‐circ_0012856 + miR‐124 inhibitor group (lentiviral infection with sh‐circ_0012856 and miR‐124 inhibitor); sh‐NC + oe‐NC group (lentiviral infection with sh‐NC and oe‐NC); sh‐circ_0012856 + oe‐NC group (lentiviral infection with sh‐circ_0012856 and oe‐NC); and sh‐circ_0012856 + oe‐STAT3 group (lentiviral infection with sh‐circ_0012856 and oe‐STAT3).

### 2.8 FISH technique

The subcellular localization of circ_0012856 was identified using fluorescence in situ hybridization (FISH) technology. The procedure followed the Ribo^TM^ lncRNA FISH Probe Mix (Red) (C10920, RiboBio, China) protocol, as outlined below: Cells were seeded onto glass coverslips placed in a 24‐well plate at a density of 6 × 10^4^ cells/well, achieving 60%–70% confluence. The cells were fixed with 1 mL of 4% paraformaldehyde at room temperature for 10 min, followed by washing. Each well was then incubated with 1 mL of pre‐cooled permeabilization solution (phosphate‐buffered saline, PBS, containing 0.5% Triton X‐100) at 4°C for 5 min, followed by washing. Next, 200 μL of pre‐hybridization solution was added to each well and incubated at 37°C for 30 min. After removing the pre‐hybridization solution, 250 μL of hybridization solution containing circ_0012856 probe (300 ng/mL) (Eurogentec) was added to each well, and the plate was incubated overnight at 37°C in the dark. All subsequent steps were also carried out in the dark. Cells were washed three times at 42° C with washing solutions in the following order: Wash I (4×SSC, 0.1% Tween‐20), Wash II (2×SSC), Wash III (1×SSC), and 1×PBS, each for 5 min. DAPI staining solution (1:800) was applied for 10 min, followed by washing. Finally, the coverslips were mounted with nail polish, and the cells were observed under a fluorescence microscope (Olympus, Japan). Images were captured from five different fields of view.

### 2.9 Dual‐luciferase reporter assay

1 × 10^5^ cells were seeded into 24‐well plates and incubated for 24 h until the confluence reached 60%–70%. Preconstructed luciferase reporter plasmids, including circ_0012856 wild‐type (WT), circ_0012856 mutant (MUT), EZH2 3′‐UTR WT, EZH2 3′‐UTR MUT, STAT3 3′‐UTR WT, and STAT3 3′‐UTR MUT, were provided by RiboBio, China. Using Lipofectamine 3000 reagent (L3000150, Invitrogen, USA), the reporter plasmids were co‐transfected with NC mimic or miR‐124 mimic (purchased from RiboBio, China) into HEK293T cells. After 48 h, the luciferase activity of both firefly and Renilla luciferase was measured using the Dual‐Luciferase Reporter Assay System (CAS: E1910, Promega, USA) and recorded with a GloMax 96 microplate luminometer (Promega, USA). The experiment was repeated three times.

### 2.10 RNA pull‐down

Biotin‐labeled probes, including Bio‐NC, Bio‐circ_0012856 WT, and Bio‐circ_0012856 MUT, were synthesized by Guangzhou RiboBio Co., Ltd. Streptavidin magnetic beads (434302, Life Technologies) were incubated with the probes at 25° C for 2 h. The probe‐coated beads were then incubated with the cell lysate overnight at 4°C. After incubation, the RNA‐protein complexes were eluted from the beads and purified using a Trizol reagent (15596026, Invitrogen, Thermo Fisher Scientific, USA). Finally, the enrichment of miR‐124 by the circ_0012856 probe was quantified using qRT‐PCR.

### 2.11 CCK‐8 assay

The cell counting Kit‐8 (CCK‐8) assay kit (Beyotime, C0037, Shanghai, China) was used to assess cell viability. Transfected HT22 cells were seeded into 96‐well plates at a density of 1 × 10^4^ cells per well. After 24 h of incubation, 10 μL of CCK‐8 reagent was added to each well, followed by a 2‐h incubation. The optical density (OD) was then measured at 450 nm using a microplate reader (Bio‐Rad, USA).

### 2.12 Flow cytometry

According to the manufacturer's instructions, neuronal apoptosis was analyzed using the Annexin V‐FITC/PI apoptosis detection kit (CA1020, Solarbio). Treated cells were collected and resuspended in a binding buffer, followed by incubation with Annexin V‐FITC and PI dyes for 15 min at room temperature in the dark. Apoptosis was then assessed using a flow cytometer (BD Biosciences, USA), and the percentage of apoptotic cells was calculated using flow cytometry analysis software.

### 2.13 Detection of autophagic flux using mCherry‐GFP‐LC3 dual fluorescence reporter system

HT22 cells were seeded on 35 mm confocal dishes, reaching approximately 70% confluence, and were then infected with AD‐mCherry‐GFP‐LC3 adenovirus (C3011, Beyotime) at a MOI of 20. The cells were incubated at 37°C for 6 h. After infection, the cells were fixed with 4% paraformaldehyde for 10 min and permeabilized with 0.15% Triton X‐100 (ST1722, Beyotime) for 10 min. The fixed cells were incubated with DAPI (C1005, Beyotime, Shanghai, China) for 5 min, followed by three washes with PBS. Finally, the autophagic flux was observed under a confocal microscope (Leica, Germany), and the autophagic activity was assessed by analyzing the changes in mCherry and GFP signals.

### 2.14 Transmission electron microscopy (TEM)

For in vivo experiments, mouse distal sciatic nerve tissues were cut into 1 mm^3^ blocks and fixed in 2% glutaraldehyde. For in vitro experiments, cells were harvested and washed with PBS; then, the cell pellets were fixed in 2% glutaraldehyde. After sectioning and double staining with uranyl acetate and lead citrate, the samples were observed and imaged using a TEM system (HT7800, Hitachi, Japan).

### 2.15 Immunofluorescence staining

Cells were routinely digested, counted, and seeded into immunofluorescence chambers at a density of 2 × 10^5^ cells per well. When the cell confluence reached approximately 90%, the cells were washed three times with PBS (on ice). Cells were then fixed with 4% paraformaldehyde (P0099, Beyotime), with 1 mL added per well, and incubated at room temperature for 15 min. After washing three times with PBS, 5% BSA (37520, Thermo) was added for blocking, and the cells were incubated for 30 min. Primary antibodies, including LC3 (1:200, ab192890, UK), P62 (1:200, ab109012, UK), iNOS (1:200, ab178945, UK), and Arg1 (1:200, ab315110, UK), were diluted in BSA and incubated overnight at 4°C. After washing with PBS three times, secondary antibodies, Goat anti‐rabbit IgG H&L (FITC) (ab6717, Abcam, UK) or Goat anti‐rabbit IgG H&L (Alexa Fluor® 647) (ab150083, Abcam, UK), were added and incubated for 1 h at room temperature in the dark. The cells were washed thrice with PBS and stained with DAPI (C1005, Beyotime, Shanghai, China) for 15 min in the dark. A fluorescence mounting medium was added after three more washes with PBS in the dark. The cells were observed and imaged using a confocal microscope (LSM 800, Zeiss, Germany), and quantitative analysis was performed using Image‐Pro Plus 6.0 software.

### 2.16 RT‐qPCR detection

Total RNA was extracted from cells using Trizol reagent (15596026, Invitrogen, Thermo Fisher Scientific, USA), and the concentration and purity of the extracted RNA were measured using a Nanodrop2000 UV spectrophotometer (1011U, Nanodrop, USA). The RNA was reverse‐transcribed into cDNA following the PrimeScript RT reagent Kit instructions (RR047A, Takara, Japan). The reverse transcription conditions were set at 37°C for 30–50 min, followed by 85°C for 5 s. RT‐qPCR was performed using the Fast SYBR Green PCR kit (RR820A, Takara, Japan) and the ABI PRISM 7300 RT‐PCR system (Applied Biosystems, Thermo Fisher Scientific, USA). The reaction conditions were 95°C for 5 min for pre‐denaturation, followed by 40 cycles of 95°C for 30 s for denaturation, 57°C for 30 s for annealing, and 72°C for 30 s for extension. Each well was tested in triplicate. GAPDH was used as an internal control for circRNA and mRNA, whereas U6 was used as an internal control for miRNA. The relative gene expression was analyzed using the 2^−ΔΔCt^ method, where ΔΔCt = (Ct value of the target gene in the experimental group—Ct value of the housekeeping gene in the experimental group)—(Ct value of the target gene in the control group—Ct value of the housekeeping gene in the control group). The experiment was repeated three times. Primer sequences are listed in Table S1, and the primers were synthesized by Sangon Biotech (Shanghai, China).

### 2.17 Western blot (WB)

Tissues or cells were collected and lysed using enhanced RIPA lysis buffer (P0013B, Beyotime Biotechnology Co., Ltd., Shanghai) containing protease inhibitors to extract the total protein. Protein concentrations were determined using the BCA protein quantification kit (P0012, Beyotime). The loading volume was adjusted to 30 μg of protein per lane with deionized water. A 12.5% SDS‐PAGE separating gel and a stacking gel were prepared. Protein samples were mixed with loading buffer, boiled at 100°C for 5 min, cooled on ice, and centrifuged. Equal amounts of protein were loaded into each lane using a micropipette for electrophoresis. After electrophoresis, the proteins were transferred onto a PVDF membrane. The PVDF membrane was blocked with 5% nonfat milk at 4°C overnight. The following primary antibodies were added at a dilution of 1:1000: iNOS (ab178945, Abcam, UK), TNF‐α (ab183218, Abcam, UK), Arg1 (ab315110, Abcam, UK), CD206 (ab64693, Abcam, UK), LC3 (ab192890, Abcam, UK), P62 (ab109012, Abcam, UK), β‐actin (ab8226, Abcam, UK), and GAPDH (ab9485, Abcam, UK). The membranes were incubated with primary antibodies overnight at 4°C. After three washes with PBS at room temperature (5 min each), HRP‐conjugated goat anti‐rabbit IgG secondary antibody (1:5000, ab6721, Abcam, UK) was added, followed by incubation at 37° C for 1 h with gentle shaking. The membrane was washed thrice times with PBS at room temperature for 5 min per wash. Pierce™ ECL WB Substrate (32209, Thermo Scientific, USA) was mixed (equal parts solution A and B) and applied to the membrane in a dark room. The membrane was then exposed and imaged using a gel imaging system. The band intensities were quantified using Image J software, and the housekeeping proteins GAPDH or β‐actin were used as loading controls.

### 2.18 ELISA detection

Cell culture supernatants were collected, and mouse distal sciatic nerve tissues were homogenized with 5–10 mL of pre‐cooled PBS. The homogenate was further processed by ultrasonic disruption and centrifuged at 850×g for 15 min at 4°C to collect the supernatant for subsequent experiments. Cells were digested with trypsin, collected by centrifugation, and subjected to ultrasonic disruption. The cell lysates were centrifuged at 1500×g for 10 min at 4°C, and the supernatant was collected for further analysis. Enzyme‐linked immunosorbent assay (ELISA) assays were performed using mouse TNF‐α ELISA kit (ab208348, Abcam, UK), mouse IL‐6 ELISA kit (ab222503, Abcam, UK), and mouse IL‐10 ELISA kit (ab255729, Abcam, UK) according to the manufacturer's instructions. Monoclonal antibodies specific to IL‐6, TNF‐α, and IL‐10 were pre‐coated onto 96‐well microplates, and the plates were incubated overnight at 4°C. After blocking at room temperature for 1 h, the plates were washed with PBS and processed according to the kit instructions. The OD values were measured at 450 nm using a microplate reader (A51119500C, Thermo Fisher, USA).

### 2.19 Establishment of a diabetic mouse model

Male C57BL/6J mice (Catalog No.: 219, 18–22g, 6 weeks old) were purchased from Beijing Vital River Laboratory Animal Technology Co., Ltd. The animals were housed under controlled temperature (23 ± 2°C), humidity (50%), and a 12‐h light/dark cycle. Mice were fed a standard laboratory diet and had free access to tap water. Streptozotocin (STZ, HY‐13753) was purchased from MCE. Before STZ injection, mice were fasted for 12 h. STZ was administered via intraperitoneal injection at a 55 mg/kg dose for five consecutive days. The remaining 50 mice received intraperitoneal injections of physiological saline and served as controls. Blood glucose levels were monitored throughout the experiment using a glucose meter (Accu‐Check Performa Nano, Roche, Basel, Switzerland). Seven days after the final injection, blood glucose levels were measured, and mice with glucose concentrations greater than 16.5 mM were selected for further experiments.^25^

The mice were divided into 5 groups, with 6 mice per group: control group (untreated mice); model group (DPN mice); model + sh‐NC + NC antagomir group (DPN mice, tail vein injection of sh‐NC and NC antagomir); model + sh‐circ_0012856 + NC antagomir group (DPN mice, tail vein injection of sh‐circ_0012856 and NC antagomir); and model + sh‐circ_0012856 + miR‐124 antagomir group (DPN mice, tail vein injection of sh‐circ_0012856 and miR‐124 antagomir). One day before STZ injection, the mice were injected via the tail vein with 2 × 10^7^ TU lentivirus (sh‐NC or sh‐circ_0012856) or 5 nmol NC antagomir, miR‐124 antagomir (purchased from RiboBio). The injections were administered once every 4 days for four injections. Various functional analyses were performed on the mice 2 days after the final injection.^26^, ^27^

### 2.20 Hot plate test

The hot plate test, utilizing a hot plate apparatus (IITC Life Science, USA), was used to assess nociceptive sensitivity in mice. The right hind paw of each mouse was placed on the hot plate, maintained at 52°C ± 2°C, with a cutoff time set at 20 s. The paw withdrawal latency of the right hind paw was recorded.

### 2.21 Mechanical allodynia test

Mechanical allodynia was assessed using von Frey filaments (Stoelting, USA). Filaments with varying pressures were applied perpendicularly to the plantar surface of the hind paw. The pressure at which the hind paw exhibited a quick withdrawal response due to pain was recorded. The plantar surface was stimulated no more than six times per minute to prevent accidental tissue damage.

### 2.22 Toluidine blue staining

Fixed mouse distal sciatic nerve tissues were embedded in paraffin and sectioned at a thickness of 1 μm. The sections were fixed in 10% paraformaldehyde solution (A500684‐0500, Sangon Biotech) for 30 min, followed by three washes with PBS. The samples were then immersed in 0.1% toluidine blue solution (E670105‐0100, Sangon Biotech) for approximately 15 min for staining. After staining, the samples were washed several times with PBS. The stained sections were placed on microscope slides, a drop of mounting medium was added, and a coverslip was applied for observation. Histopathological changes in the tissues were examined using a light microscope (Olympus, Japan).

### 2.23 Statistical analysis

Data were derived from at least three independent experiments and are presented as the mean ± standard deviation (Mean ± SD); for comparisons between two groups, an independent‐sample *t*‐test was used. For comparisons among three or more groups, a one‐way analysis of variance (ANOVA) was employed. If ANOVA indicated significant differences, Tukey's HSD post hoc test was conducted to assess pairwise group differences. The Mann–Whitney *U* test or Kruskal–Wallis *H* test was used for data that did not meet the assumptions of normality or homogeneity of variance. All statistical analyses were performed using GraphPad Prism 9 (GraphPad Software, Inc.) and R. A significance level of 0.05 was applied to all tests, and two‐sided *p*‐values less than 0.05 were considered statistically significant.

## 3 RESULTS

### 3.1 Identification of pathogenic circ_0012856 and its targeted binding miR‐124 in DPN

**FIGURE 1:**
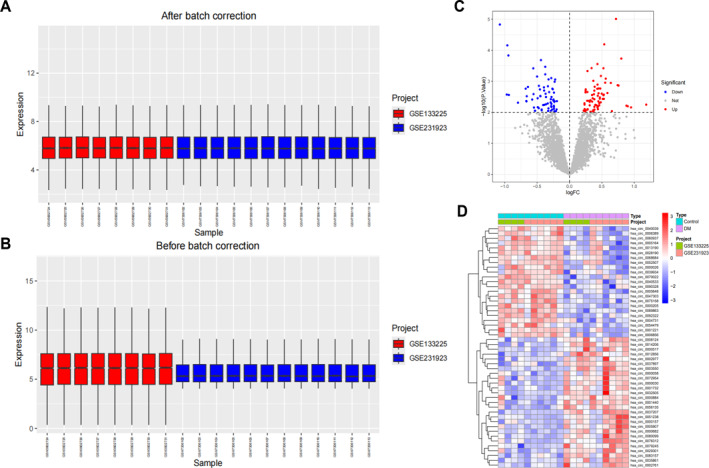
circRNA differential expression analysis. (A, B) Box plots illustrating the data distribution before and after batch effect removal; (C) volcano plot showing differentially expressed circRNAs between the control and DM groups in the merged dataset; (D) heatmap of DEcircRNAs between the control and DM groups in the merged dataset. Control: *n* = 10, DM: *n* = 10.

**FIGURE 2:**
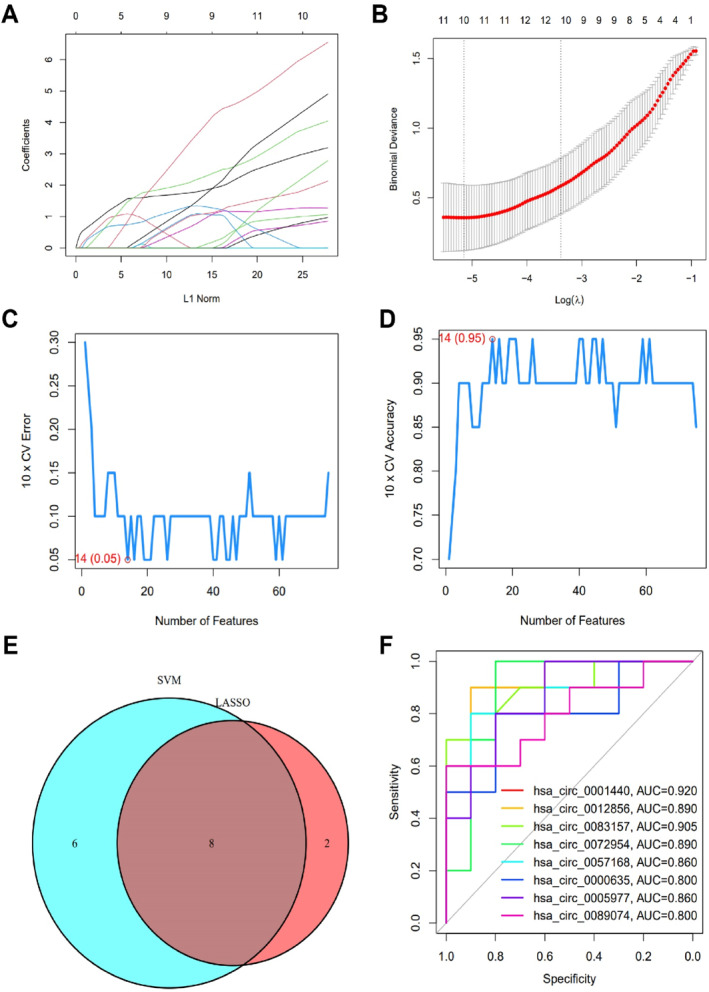
Screening of key circRNA features. (A, B) Feature circRNAs identified using the least absolute shrinkage and selection operator logistic regression algorithm with covariates selected by the regularization parameter λ; (C, D) feature circRNAs selected by the support vector machine recursive feature elimination algorithm; (E) Venn diagram showing the overlapping key feature circRNAs identified by both LASSO and SVM‐RFE; (F) receiver operating characteristic analysis of the 8 key feature circRNAs. Control: *n* = 10, DM: *n* = 10.

DPN is one of the most common and severe complications of DM, with a complex pathogenesis. Current research indicates that circRNAs play a crucial role in developing DPN.^28^ To identify the circRNAs involved in DPN pathogenesis, we integrated two DM‐related circRNA datasets, GSE133225 and GSE231923, and used the R package “sva” to eliminate batch effects (Figure 1A,B). Differential expression analysis identified 146 differentially expressed circRNAs, including 75 upregulated and 71 downregulated DEcircRNAs (Figure 1C,D). LASSO analysis of the upregulated pathogenic circRNAs revealed a set of 10 circRNAs (Figure 2A,B). Additionally, the SVM‐RFE algorithm identified 14 circRNAs (Figure 2C,D). The overlap between these two methods produced 8 circRNAs: circ_0001440, circ_0012856, circ_0083157, circ_0072954, circ_0057168, circ_0000635, circ_0005977, and circ_0089074, which were identified as key feature circRNAs (Figure 2E). ROC analysis demonstrated that all 8 circRNAs had an AUC of ≥0.8 (Figure 2F), indicating high diagnostic potential. Therefore, these 8 circRNAs were selected for further analysis as key circRNAs in our study.

**FIGURE 3:**
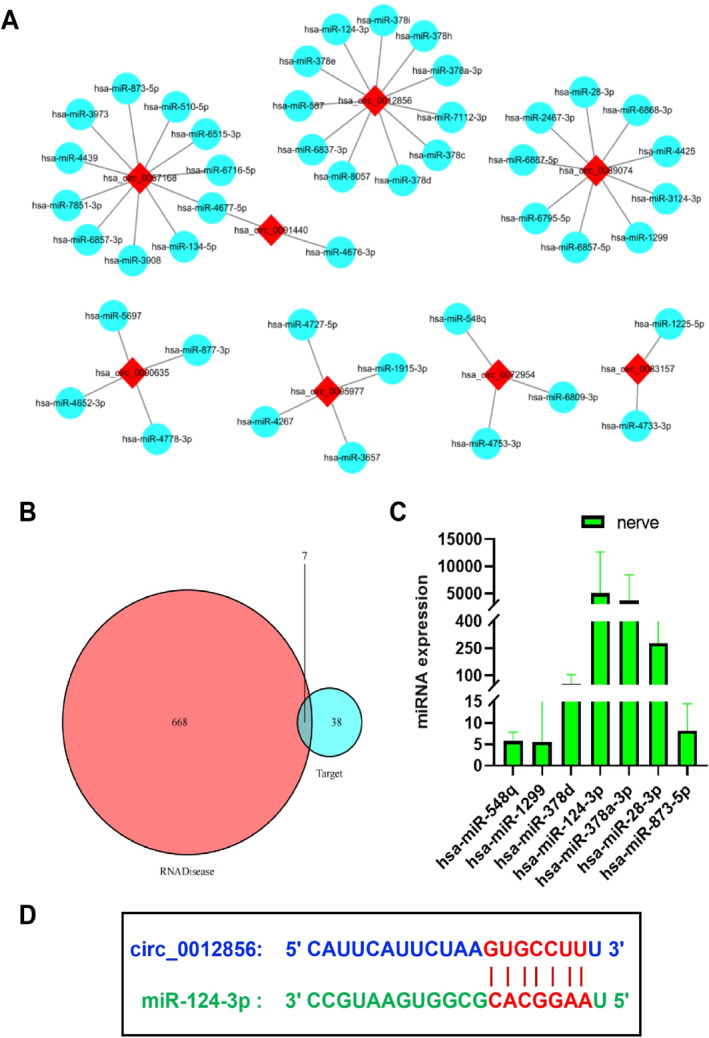
Target miRNA prediction for circ_0012856. (A) circRNA‐miRNA interaction network diagram; (B) intersection of target miRNAs for circ_0012856 predicted by circAtlas, circBank, and miRDB with DM‐related miRNAs from the RNADisease database; (C) expression of miRNAs in neurons from the TissueAtlas database; (D) potential binding sites between circ_0012856 and miR‐124. miRNAs, microRNAs.

According to the ceRNA theory, circRNAs act as sponges for miRNAs, thereby regulating miRNA expression.^29^ Using the circles, circBank, and miRDB databases, we predicted potential miRNA targets for the eight identified circRNAs, resulting in 46 circRNA‐miRNA pairs (Figure 3A). Further analysis using the RNADisease database identified 675 DM‐related miRNAs. By comparing these with the predicted target miRNAs, we narrowed down 7 candidate miRNAs: hsa‐miR‐548q, miR‐1299, miR‐378d, miR‐124‐3p, miR‐378a‐3p, miR‐28‐3p, and miR‐873‐5p (Figure 3B). Subsequent analysis of these miRNAs' expression levels in neurons revealed that miR‐124‐3p had the highest expression (Figure 3C). Moreover, previous studies have shown that miR‐124 expression is downregulated in the distal sciatic nerve tissue of diabetic mice, and increasing miR‐124 levels can alleviate the progression of DPN.^30^ Based on this, we selected miR‐124 and its interacting circRNA, circ_0012856, for further investigation. Figure 3D illustrates the potential binding sites between circ_0012856 and miR‐124.

In conclusion, we successfully identified circ_0012856 as a pathogenic circRNA in DPN, which exerts its effect by sponging miR‐124. This discovery provides a novel potential target for diagnosing and treating DPN.

### 3.2 Bioinformatics analysis results show that miR‐124 participates in DPN by activating EZH2/STAT3

**FIGURE 4:**
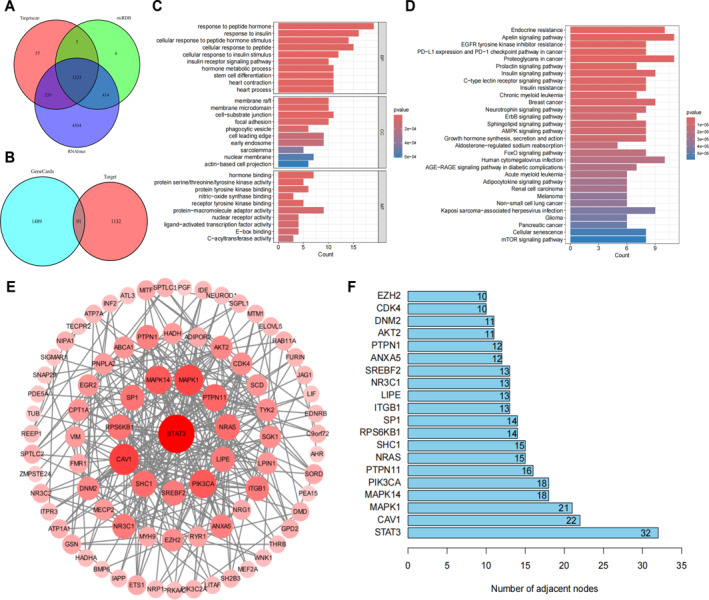
Target gene prediction for miR‐124. (A) Intersection of potential target genes for miR‐124 predicted by TargetScan, RNAInter, and miRDB databases; (B) intersection of potential target genes for miR‐124 with DPN‐related genes obtained from GeneCards; (C, D) Gene ontology and Kyoto encyclopedia of genes and genomes analysis results of the intersecting genes; (E) protein–protein interaction network diagram of the intersecting genes; (F) bar chart of the top 20 hub genes in the PPI network.

Studies have shown that miRNAs regulate the expression of target genes by degrading mRNA or inhibiting translation.^31^ To further investigate the mechanism of miR‐124 in DPN, we predicted its potential target genes using the TargetScan, RNAInter, and miRDB databases (Figure 4A) and performed an intersection analysis with DPN‐related genes from the GeneCards database, identifying 91 candidate genes (Figure 4B). GO and KEGG functional enrichment analysis revealed that these genes are primarily associated with insulin resistance, neurotrophin signaling, AMPK, and mTOR signaling pathways (Figure 4C,D).

**FIGURE 5:**
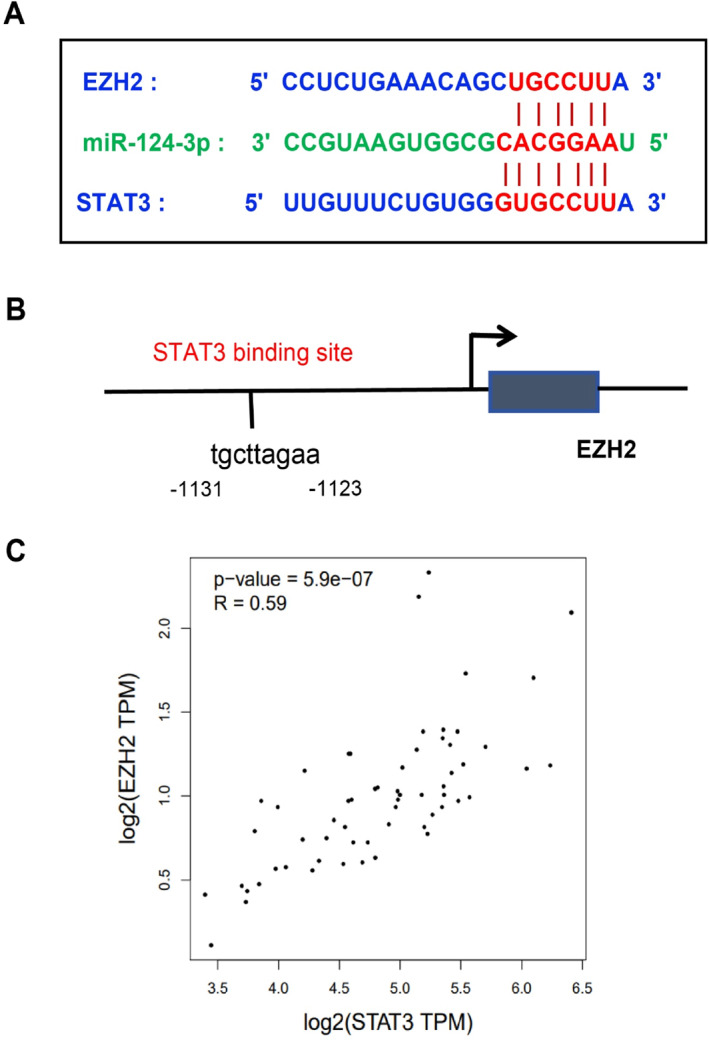
Interaction analysis between STAT3 and EZH2. (A) Potential binding sites of miR‐124 with STAT3 and EZH2; (B) potential STAT3 binding sites on the EZH2 promoter; (C) correlation analysis between STAT3 and EZH2 in brain and spinal cord tissues based on the GEPIA database.

Further protein interaction network analysis identified STAT3 as the most highly connected hub gene among the top 20 hub genes (Figure 4E,F). Studies have demonstrated that miR‐124 can inhibit DPN progression by downregulating EZH2 and STAT3, reducing cell apoptosis and enhancing Schwann cell autophagy.^30^ Protein interaction analysis revealed a functional relationship between STAT3 and EZH2. Figure 5A illustrates the potential binding sites of miR‐124 with EZH2 and STAT3. Additionally, JASPAR database analysis identified a potential STAT3 binding site in the EZH2 promoter (Figure 5B). Correlation analysis further confirmed a significant positive relationship between STAT3 and EZH2 expression in brain and spinal cord tissues (*R* = 0.59, *p* = 5.9e‐07) (Figure 5C).

These results suggest that circ_0012856 may play a key role in DPN by competitively binding to miR‐124, thereby regulating the expression of EZH2 and STAT3. This mechanism could contribute to the pathogenesis of DPN.

### 3.3 Validation of circ_0012856's role in regulating DPN progression via competitive binding to miR‐124 and upregulating EZH2/STAT3

**FIGURE 6:**
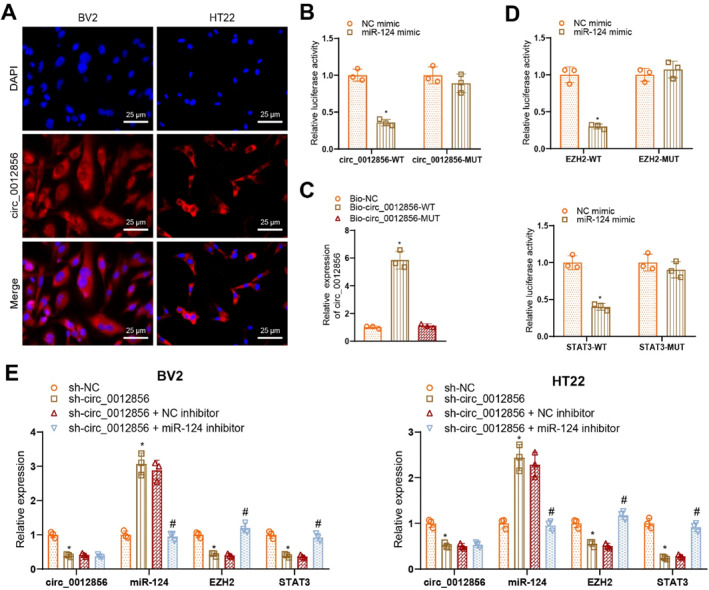
circ_0012856 regulates EZH2 and STAT3 expression by sponging miR‐124. (A) Fluorescence in situ hybridization showing the localization of circ_0012856 in BV2 microglial cells and HT22 neuronal cells (scale bar: 25 μm); (B) luciferase reporter assay verifying the interaction between circ_0012856 and miR‐124; (C) RNA pull‐down assay confirming the binding between circ_0012856 and miR‐124; (D) luciferase reporter assay validating the regulatory effects of miR‐124 on EZH2 and STAT3; (E) RT‐qPCR analysis of circ_0012856, miR‐124, EZH2, and STAT3 expression in BV2 microglial cells and HT22 neuronal cells in different groups. *p < 0.05 compared with the NC mimic group, Bio‐NC group, or sh‐NC group; #*p* < 0.05 compared with the sh‐circ_0012856 + NC inhibitor group. Cell experiments were repeated three times.

Based on the previous bioinformatic analysis, we hypothesized that circ_0012856 may regulate the expression of EZH2 and STAT3 in DPN by competitively binding to miR‐124. To test this hypothesis, we performed in situ hybridization (ISH) to determine the cellular localization of circ_0012856. The results indicated that circ_0012856 is primarily localized in the cytoplasm of BV2 microglial cells and HT22 neurons (Figure 6A). Next, we constructed WT and MUT 3′UTR luciferase reporter vectors containing the binding sites for circ_0012856 and co‐transfected them with miR‐124 overexpression plasmids into HEK293T cells. Dual‐luciferase assay results showed that in cells co‐transfected with the WT circ_0012856 (circ_0012856‐WT), the luciferase activity was reduced in the miR‐124 mimic group compared to the NC mimic group, whereas no significant change was observed in the MUT (circ_0012856‐MUT) group (Figure 6B). Furthermore, RNA pull‐down assays confirmed the direct interaction between circ_0012856 and miR‐124. The results demonstrated that Bio‐circ_0012856‐WT enriched miR‐124 compared to Bio‐NC and Bio‐circ_0012856‐MUT (Figure 6C). These findings confirm that circ_0012856 competitively binds to miR‐124 and regulates its activity.

We further constructed WT and MUT 3′UTR luciferase reporter vectors containing the binding sites for EZH2 and STAT3 and co‐transfected them with miR‐124 overexpression plasmids into HEK293T cells. The dual‐luciferase assay results showed that, compared to the NC mimic group, the luciferase activity was reduced in the miR‐124 mimic group co‐transfected with the WT EZH2 and STAT3 3′UTR reporter vectors, whereas no significant change in luciferase activity was observed in the MUT 3′UTR reporter groups (Figure 6D).

Using RT‐qPCR, we measured circ_0012856, miR‐124, EZH2, and STAT3 expression levels in BV2 microglial cells and HT22 neuronal cells across different groups. The results showed that compared to the sh‐NC group, circ_0012856, EZH2, and STAT3 expressions were reduced in the sh‐circ_0012856 group, whereas miR‐124 expression was increased. Furthermore, in the sh‐circ_0012856 + miR‐124 inhibitor group, there was no significant difference in circ_0012856 expression compared to the sh‐circ_0012856 + NC inhibitor group, but miR‐124 expression increased, and the expression of EZH2 and STAT3 decreased (Figure 6E).

These results suggest that miR‐124 can inhibit the expression of EZH2 and STAT3 through its interactions, whereas circ_0012856 upregulates the expression of EZH2 and STAT3 by competitively binding to miR‐124.

### 3.4 circ_0012856 inhibits neuronal autophagy via miR‐124‐mediated upregulation of EZH2

**FIGURE 7:**
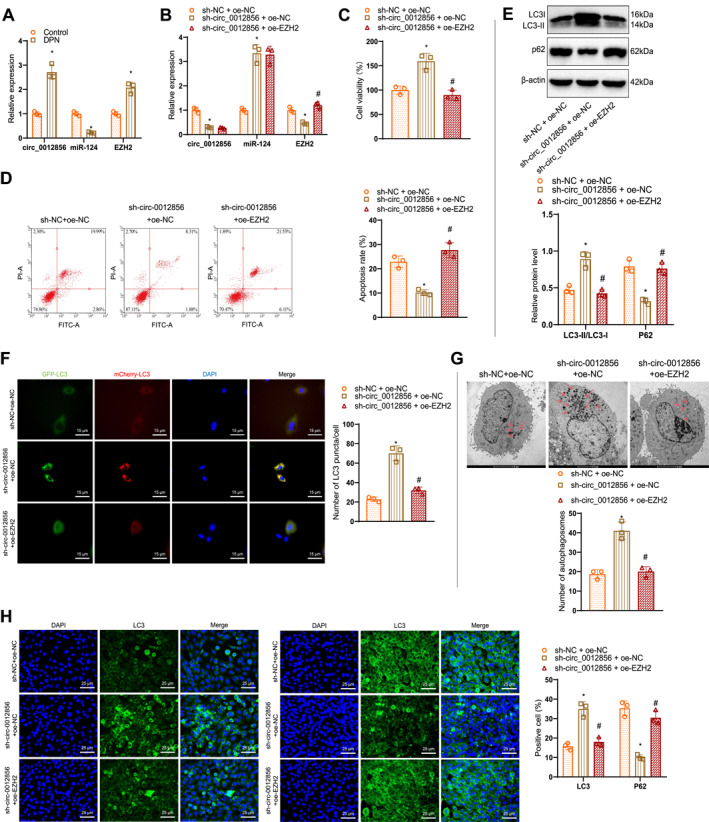
circ_0012856 inhibits neuronal autophagy by upregulating EZH2 through miR‐124. (A) RT‐qPCR analysis of circ_0012856, miR‐124, and EZH2 expression in different treatment groups; (B) RT‐qPCR analysis of circ_0012856, miR‐124, and EZH2 expression in different treatment groups; (C) CCK‐8 assay to measure cell viability in different treatment groups; (D) flow cytometry analysis of apoptosis rates in different treatment groups; (E) western blot analysis of LC3‐II and p62 expression levels in different treatment groups; (F) fluorescence microscopy showing autophagic flux using the mCherry‐GFP‐LC3 dual fluorescence reporter system (scale bar: 15 μm); (G) transmission electron microscopy showing changes in autophagosomes in different treatment groups (scale bar: 5 μm); (H) immunofluorescence staining showing LC3 and p62 fluorescence intensity in different treatment groups (scale bar: 25 μm). *p < 0.05 compared with the control group or the sh‐NC + oe‐NC group; #*p* < 0.05 compared with the sh‐circ_0012856 + oe‐NC group. Cell experiments were repeated three times.

The in vitro experiments confirmed that circ_0012856 upregulates EZH2 expression by competitively binding to miR‐124. Previous studies have shown that a high‐glucose environment promotes the expression of histone methyltransferase EZH2, and elevated EZH2 levels inhibit the synthesis of LC3, a key autophagy marker. Treatment with EZH2‐specific inhibitors (EPZ005687, EI1, GSK126, GSK343, and UNC1999) promotes LC3 synthesis and autophagy.^32^ These findings suggest that in DPN, circ_0012856 may suppress autophagy by upregulating EZH2 through competitive binding to miR‐124, thereby inhibiting high‐glucose‐induced neuronal damage. In an in vitro DPN neuronal cell model, the results showed that, compared to the control group, the expression of circ_0012856 and EZH2 was upregulated, whereas miR‐124 levels were downregulated in the DPN group (Figure 7A).

Using shRNA or overexpression lentiviral infection in HT22 neuronal cells, the cells were divided into the following groups: sh‐NC + oe‐NC, sh‐circ_0012856 + oe‐NC, and sh‐circ_0012856 + oe‐EZH2. RT‐qPCR results showed that, compared to the sh‐NC + oe‐NC group, the expression of circ_0012856 was reduced, miR‐124 expression was increased, and EZH2 expression was decreased in the sh‐circ_0012856 + oe‐NC group. However, compared to the sh‐circ_0012856 + oe‐NC group, the sh‐circ_0012856 + oe‐EZH2 group showed no significant difference in circ_0012856 and miR‐124 expression, whereas EZH2 expression was increased (Figure 7B).

In the DPN in vitro HT22 neuronal cell model, cell viability was assessed using the CCK‐8 assay. The results showed that, compared to the sh‐NC + oe‐NC group, cell viability in the sh‐circ_0012856 + oe‐NC group was increased. However, compared to the sh‐circ_0012856 + oe‐NC group, cell viability was reduced in the sh‐circ_0012856 + oe‐EZH2 group (Figure 7C). Flow cytometry was used to assess apoptosis rates, and the results indicated that apoptosis was reduced in the sh‐circ_0012856 + oe‐NC group compared to the sh‐NC + oe‐NC group, whereas apoptosis was notably increased in the sh‐circ_0012856 + oe‐EZH2 group compared to the sh‐circ_0012856 + oe‐NC group (Figure 7D).

WB analysis showed that in HT22 neurons, compared to the sh‐NC + oe‐NC group, the LC3‐II/LC3I ratio was increased, and p62 expression was decreased in the sh‐circ_0012856 + oe‐NC group. In contrast, compared to the sh‐circ_0012856 + oe‐NC group, the LC3‐II/LC3I ratio was decreased, and p62 expression was increased in the sh‐circ_0012856 + oe‐EZH2 group (Figure 7E). Autophagic flux, assessed using the mCherry‐GFP‐LC3 dual‐fluorescent reporter system, showed a significant enhancement in autophagy in the sh‐circ_0012856 + oe‐NC group compared to the sh‐NC + oe‐NC group, whereas autophagic flux was markedly reduced in the sh‐circ_0012856 + oe‐EZH2 group compared to the sh‐circ_0012856 + oe‐NC group (Figure 7F). TEM further confirmed an increase in autophagosomes in the sh‐circ_0012856 + oe‐NC group, whereas EZH2 overexpression reduced autophagosomes (Figure 7G). Immunofluorescence staining revealed that, compared to the sh‐NC + oe‐NC group, LC3 fluorescence intensity was enhanced, and p62 fluorescence intensity was reduced in the sh‐circ_0012856 + oe‐NC group, whereas in the sh‐circ_0012856 + oe‐EZH2 group, LC3 fluorescence intensity was decreased and p62 fluorescence intensity was increased (Figure 7H).

The results indicate that silencing circ_0012856 regulates miR‐124 to enhance neuronal viability, inhibit apoptosis, and promote autophagy. However, EZH2 overexpression can reverse the effects of circ_0012856 silencing on neuronal viability and autophagy, highlighting EZH2's critical role in modulating these processes.

### 3.5 circ_0012856 upregulates STAT3 via miR‐124 to promote M1 polarization of microglia

During the development of DPN, activated microglia undergo M1 polarization, which promotes the secretion of inflammatory cytokines and exacerbates inflammation in the surrounding neural tissues, accelerating myelin sheath degeneration. This process is closely linked to activating the pro‐inflammatory JAK2/STAT3 signaling pathway. STAT3 activation drives the transformation of microglia/macrophages to the M1 phenotype, and inhibiting STAT3 can partially reverse macrophage polarization under stress conditions.^33^

**FIGURE 8:**
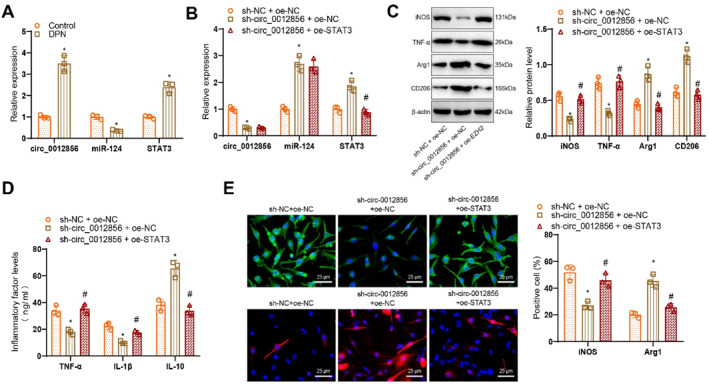
circ_0012856 promotes the M1 polarization of microglia by upregulating STAT3 through miR‐124. (A) RT‐qPCR analysis of circ_0012856, miR‐124, and EZH2 expression in different treatment groups; (B) RT‐qPCR analysis of circ_0012856, miR‐124, and STAT3 expression in different treatment groups; (C) western blot analysis of M1 and M2 marker expression levels in different treatment groups; (D) ELISA measurement of TNF‐α, IL‐1β, and IL‐10 concentrations in the cell culture supernatant of different treatment groups; (E) immunofluorescence staining showing the fluorescence intensity of iNOS and Arg1 in different treatment groups (scale bar: 25 μm). *p < 0.05 compared with the sh‐NC + oe‐NC group; #*p* < 0.05 compared with the sh‐circ_0012856 + oe‐NC group. Cell experiments were repeated three times.

In an in vitro DPN model using BV2 microglial cells, the results showed that compared to the control group, the DPN group exhibited upregulated expression of circ_0012856 and EZH2, whereas miR‐124 levels were downregulated (Figure 8A). Microglial cells were transfected with either shRNA or overexpression plasmids and divided into the following groups: sh‐NC + oe‐NC, sh‐circ_0012856 + oe‐NC, and sh‐circ_0012856 + oe‐STAT3. RT‐qPCR results demonstrated that, compared to the sh‐NC + oe‐NC group, circ_0012856 expression was reduced, miR‐124 expression increased, and STAT3 expression decreased in the sh‐circ_0012856 + oe‐NC group. However, in the sh‐circ_0012856 + oe‐STAT3 group, no significant differences in circ_0012856 or miR‐124 expression were observed compared to the sh‐circ_0012856 + oe‐NC group, whereas STAT3 expression was increased (Figure 8B).

WB analysis revealed that, compared to the sh‐NC + oe‐NC group, the expression of M1 markers iNOS and TNF‐α was reduced, whereas M2 markers, Arg1 and CD206, were increased in microglial cells from the sh‐circ_0012856 + oe‐NC group. In contrast, compared to the sh‐circ_0012856 + oe‐NC group, the sh‐circ_0012856 + oe‐STAT3 group showed increased expression of M1 markers, iNOS and TNF‐α, and decreased expression of M2 markers, Arg1 and CD206 (Figure 8C). ELISA results further confirmed that, compared to the sh‐NC + oe‐NC group, the concentrations of TNF‐α and IL‐1β in the culture supernatant of microglial cells were reduced, whereas IL‐10 concentration was increased in the sh‐circ_0012856 + oe‐NC group. However, in the sh‐circ_0012856 + oe‐STAT3 group, the concentrations of TNF‐α and IL‐1β were increased, and IL‐10 concentration was reduced compared to the sh‐circ_0012856 + oe‐NC group (Figure 8D).

Immunofluorescence staining showed that, compared to the sh‐NC + oe‐NC group, iNOS fluorescence intensity was reduced, whereas Arg1 fluorescence intensity was increased in microglial cells from the sh‐circ_0012856 + oe‐NC group. In contrast, compared to the sh‐circ_0012856 + oe‐NC group, the sh‐circ_0012856 + oe‐STAT3 group showed a significant increase in iNOS fluorescence intensity and a decrease in Arg1 fluorescence intensity (Figure 8E).

These results suggest that silencing circ_0012856 regulates miR‐124 to promote M1 polarization of microglial cells. However, overexpression of EZH2 can reverse the effect of circ_0012856 silencing on enhancing M1 polarization in microglial cells.

### 3.6 Silencing circ_0012856 promotes autophagy and inhibits M1 polarization of microglia via the miR?124/EZH2/STAT3 pathway, alleviating diabetic neuropathy

A diabetic Sprague Dawley mouse model was successfully induced through the intraperitoneal injection of STZ. Random blood glucose measurements consistently exceeding 16.7 mmol/L on three occasions confirmed the successful establishment of the diabetic model. The diabetic mice were then randomly divided into the following groups: control, model, model + sh‐NC + NC antagomir, model + sh‐circ_0012856 + NC antagomir, and model + sh‐circ_0012856 + miR‐124 antagomir. We subsequently tracked and recorded body weight changes in each group to evaluate the therapeutic effects of circ_0012856 silencing. The body weight tracking data (Table S2) showed that mice in the model group exhibited significant weight loss, consistent with the catabolic effects of sustained hyperglycemia (>16.7 mM), such as muscle wasting and fat breakdown. Silencing circ_0012856 (model + sh‐circ_0012856 + NC antagomir group) partially alleviated the weight loss, possibly by improving glucose metabolism or reducing inflammation. However, the concurrent inhibition of miR‐124 (model + sh‐circ_0012856 + miR‐124 antagomir group) appeared to counteract the metabolic benefits conferred by circ_0012856 silencing, resulting in body weight decline similar to the model group.

**FIGURE 9:**
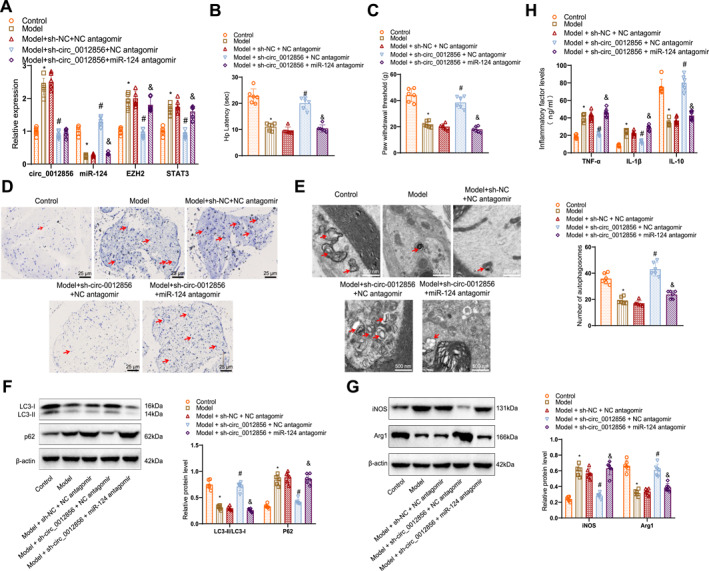
Silencing circ_0012856 promotes autophagy and inhibits microglial M1 polarization via the miR‐124/EZH2/STAT3 axis, alleviating diabetic neuropathy. (A) RT‐qPCR analysis of circ_0012856, miR‐124, EZH2, and STAT3 expression in the distal sciatic nerves of different groups of mice; (B) hot plate test measuring the right hind paw response time in each group of mice; (C) mechanical test assessing the pressure applied to the right hind paw withdrawal in each group of mice; (D) toluidine blue staining showing representative cross‐sections of the nerves in each group of mice, with arrows indicating myelin infoldings into nerve fibers (scale bar: 25 μm); (E) transmission electron microscopy showing changes in autophagosome numbers (scale bar: 500 nm); (F) WB analysis of LC3‐II and p62 expression in the distal sciatic nerve tissues of mice; (G) WB analysis of iNOS and Arg1 expression in the distal sciatic nerve tissues of mice; (H) ELISA measurement of TNF‐α, IL‐1β, and IL‐10 concentrations in the distal sciatic nerve tissues of mice. WB, western blot. *p < 0.05 compared with the control group; #*p* < 0.05 compared with the model + sh‐NC + NC antagomir group; &*p* < 0.05 compared with the model + sh‐circ_0012856 + NC antagomir group, *N* = 6.

RT‐qPCR results showed that, compared to the control group, the expression of circ_0012856, EZH2, and STAT3 was increased, whereas miR‐124 expression was decreased in the distal sciatic nerve tissues of the model group. In the model + sh‐circ_0012856 + NC antagomir group, circ_0012856, EZH2, and STAT3 expression levels were reduced, and miR‐124 expression was increased compared to the model + sh‐NC + NC antagomir group. However, in the model + sh‐circ_0012856 + miR‐124 antagomir group, circ_0012856 expression showed no significant difference compared to the model + sh‐circ_0012856 + NC antagomir group, whereas EZH2 and STAT3 expression levels were increased, and miR‐124 expression was decreased (Figure 9A).

After 8 weeks, the hot plate and mechanical allodynia test results showed that, compared to the control group, the model group exhibited reduced right hind paw response time and decreased pressure applied during paw withdrawal, indicating increased pain sensitivity. Compared to the model + sh‐NC + NC antagomir group, the model + sh‐circ_0012856 + NC antagomir group showed an increase in response time and applied pressure during paw withdrawal, reflecting a significant reduction in pain sensitivity. However, compared to the model + sh‐circ_0012856 + NC antagomir group, the model + sh‐circ_0012856 + miR‐124 antagomir group exhibited decreased response time and applied pressure during paw withdrawal, indicating a significant increase in pain sensitivity (Figure 9B,C).

Histological analysis revealed significant pathological changes in the distal sciatic nerves of the model group, including nerve fiber degeneration and infiltration of inflammatory cells. These pathological changes were markedly alleviated in the circ_0012856 silencing group, whereas the circ_0012856 + miR‐124 silencing group displayed pathological changes similar to those observed in the model group (Figure 9D). TEM showed a noticeable reduction in autophagosomes in the distal sciatic nerves of the model group. In contrast, the circ_0012856 silencing group (model + sh‐circ_0012856 + NC antagomir) exhibited a significant increase in autophagosome numbers compared to the model + sh‐NC + NC antagomir group. However, in the circ_0012856 + miR‐124 silencing group, the number of autophagosomes returned to comparable levels in the model group (Figure 9E).

WB analysis showed that, compared to the control group, the model group had reduced expression of LC3‐II/LC3‐I and p62, increased expression of iNOS, and decreased expression of Arg1 in the distal sciatic nerve tissues. Compared to the model + sh‐NC + NC antagomir group, the model + sh‐circ_0012856 + NC antagomir group exhibited increased LC3‐II expression, reduced p62 expression, decreased iNOS expression, and elevated Arg1 expression. However, compared to the model + sh‐circ_0012856 + NC antagomir group, the model + sh‐circ_0012856 + miR‐124 antagomir group showed a significant decrease in LC3‐II/LC3‐I expression, increased p62 expression, and higher iNOS expression, with reduced Arg1 expression (Figure 9F,G).

Additionally, ELISA results showed that, compared to the control group, the concentrations of inflammatory cytokines, TNF‐α and IL‐1β, were elevated, whereas the concentration of the anti‐inflammatory cytokine IL‐10 was reduced in the distal sciatic nerve tissues of the model group. Compared to the model + sh‐NC + NC antagomir group, the model + sh‐circ_0012856 + NC antagomir group exhibited lower levels of TNF‐α and IL‐1β and higher levels of IL‐10. However, compared to the model + sh‐circ_0012856 + NC antagomir group, the model + sh‐circ_0012856 + miR‐124 antagomir group showed a significant increase in TNF‐α and IL‐1β levels and a decrease in IL‐10 concentration (Figure 9H).

The above results indicate that silencing circ_0012856 alleviates diabetic neuropathy by promoting autophagy through the miR‐124/EZH2/STAT3 pathway.

## 4 DISCUSSION

This study, through both in vitro and in vivo experiments, is the first to reveal the critical role of circ_0012856 in the pathogenesis of DPN. The findings demonstrate that circ_0012856 acts as a molecular sponge for miR‐124, regulating the expression of EZH2 and STAT3, which in turn inhibit neuronal autophagy and promote microglial M1 polarization. These processes collectively exacerbate the pathological progression of DPN. This study systematically investigates the molecular mechanisms of circ_0012856 in DPN for the first time, offering new perspectives and potential molecular targets for future research and therapeutic interventions.

In recent years, the regulatory role of ncRNAs in diseases like DPN has garnered increasing attention. circRNAs, due to their unique structure, exhibit high stability and specific regulatory capabilities.^34^ Studies have shown that circRNAs function as ceRNAs by sponging miRNAs, thereby regulating downstream gene expression and playing critical roles in various pathophysiological processes.^35^, ^36^ Although some research has highlighted the potential role of circRNAs in DPN, the specific regulatory mechanism of circ_0012856 remains unclear. The novelty of this study lies in its elucidation of how circ_0012856 regulates EZH2 and STAT3 expression through the ceRNA network, clarifying its role in exacerbating DPN pathology and advancing our understanding of its pathogenesis.

In our study, circ_0012856 was found to regulate EZH2, influencing the process of neuronal autophagy. As a key enzyme involved in histone methylation, EZH2 is closely linked to autophagy. In the nervous system, elevated EZH2 expression is typically associated with reduced autophagic activity, which in turn affects neuronal survival and function.^37^, ^38^ Our in vitro experiments revealed that circ_0012856 upregulates EZH2 expression, significantly inhibiting neuronal autophagic flux, and thereby reducing neuronal viability and adaptability. This aligns with previous studies, indicating that EZH2, as a crucial autophagic regulator in neuropathology, plays a role in the development of DPN. This research systematically explores for the first time the role of circ_0012856 in regulating autophagy through EZH2, highlighting the importance of autophagy in diabetic neuropathy.

Furthermore, this study revealed that circ_0012856 promotes microglial M1 polarization by upregulating STAT3 expression, which in turn enhances inflammatory responses and exacerbates neural damage. Microglia are key immune cells in the central nervous system, playing a dual role in neuroinflammation and nerve repair. M1‐polarized microglia are typically associated with increased inflammation, whereas M2 polarization is linked to inflammation suppression and tissue repair.^39^, ^40^, ^41^ Our research demonstrates that circ_0012856 drives M1 polarization through STAT3 regulation, thereby intensifying neuroinflammation in DPN. Compared to previous studies, this research further refines the molecular mechanisms underlying microglial polarization, particularly highlighting the pivotal regulatory role of STAT3 in this process.

In the in vivo experiments, we silenced circ_0012856, which significantly improved DPN symptoms in diabetic mice, including enhanced distal sciatic nerve conduction velocity, reduced hyperalgesia, and decreased neural damage. This finding suggests that circ_0012856 plays a critical role in the pathogenesis of DPN, and its regulatory pathway may serve as a novel therapeutic target for DPN. Unlike traditional drug therapies, gene regulation‐based treatment strategies offer the potential for high specificity and low side effects, particularly in the field of circRNA, where the therapeutic prospects are promising.

The role of autophagy in the nervous system has gained increasing attention, especially in neurodegenerative diseases, trauma, and metabolic disorders, where autophagy dysfunction is considered a key mechanism underlying neural damage.^42^, ^43^, ^44^ This study found that circ_0012856 inhibits neuronal autophagy by regulating EZH2, highlighting the critical role of autophagy in the progression of DPN. EZH2, as an epigenetic regulator, plays a pivotal role in various cellular functions, including proliferation, differentiation, and survival. In neurons, the overexpression of EZH2 is closely linked to autophagy inhibition, and a reduction in autophagy may decrease neuronal resilience to stress, thereby exacerbating neural injury. Therefore, future research could explore whether EZH2 inhibitors or other drugs that enhance autophagic flux can effectively alleviate DPN symptoms. Specifically, how to restore neuronal autophagy through pharmacological interventions in diabetic patients is a promising area for further investigation. Although the HT22 neuronal and BV2 microglial cell lines used in this study are well‐established models that accurately represent neuronal and microglial function in neurodegenerative processes, including diabetic and high‐fat diet‐induced neuropathies,^45^, ^46^ we acknowledge that these models have inherent limitations. Further validation in additional neuronal and microglial cell lines is essential to confirm the generalizability of our findings. Moreover, although our study systematically demonstrates that circ_0012856 regulates the EZH2/STAT3 axis via miR‐124 sponging and plays a pivotal role in DPN, certain limitations remain. Our in vivo data indicate that circ_0012856 is a necessary factor in the pathogenesis of DPN, as its silencing significantly alleviated neuropathic symptoms in diabetic mice. However, whether circ_0012856 is sufficient to induce neuropathic changes in the absence of diabetes has not yet been addressed. It remains unclear whether circ_0012856 exerts pathogenic effects only in the context of diabetes‐associated metabolic dysregulation or inflammation. Future studies could overexpress circ_0012856 in normoglycemic mice to determine whether it is sufficient to induce neuronal autophagy inhibition and microglial M1 polarization. This would help clarify its standalone pathogenic potential. Additionally, diabetes involves complex multisystem interactions, including glucotoxicity, oxidative stress, and chronic inflammation. It is possible that circ_0012856 acts in concert with other diabetes‐related factors to drive DPN progression. Therefore, understanding the independent effects of circ_0012856 and its potential crosstalk with other signaling pathways warrants further investigation. Finally, emerging studies suggest that microglial activation is highly plastic, with phenotypes existing on a continuum or in mixed states, rather than being strictly categorized into M1 or M2.^47^, ^48^ For example, under chronic inflammation or metabolic stress, microglia may co‐express both pro‐inflammatory and reparative genes, leading to intermediate or disease‐specific states. Our conclusions are based on a limited panel of markers. Future studies should incorporate multi‐omics analyses, such as single‐cell RNA sequencing, and expand the marker panel (e.g., CD86, CD206, TREM2, and TMEM119) to comprehensively assess how circ_0012856 affects microglial functional states.

This study is the first to systematically reveal the mechanistic role of circ_0012856 in DPN through the regulation of the miR‐124/EZH2/STAT3 axis. This finding not only provides new insights into the pathological mechanisms of DPN but also offers potential therapeutic targets. Therapeutic strategies targeting circ_0012856, such as silencing its expression using shRNA, could emerge as novel treatments for DPN. Additionally, regulating the expression of miR‐124, EZH2, and STAT3 may offer new directions for DPN therapy. However, there are some limitations to this study. First, the research is primarily focused on cell and animal models; further clinical studies are necessary to validate the feasibility and efficacy of these findings. Second, the precise mechanisms of circ_0012856 and other potential targets require further exploration. Looking ahead, in‐depth studies of circ_0012856 and its regulatory network, aimed at developing targeted therapeutic strategies, hold promise for providing more effective treatment options for DPN patients.

## 5 CONCLUSION

This study reveals the critical role of circ_0012856 in DPN. circ_0012856 acts as a sponge for miR‐124, reducing its expression, which in turn upregulates the expression of EZH2 and STAT3. The increased levels of EZH2 and STAT3 promote microglial M1 polarization and inhibit neuronal autophagy, thereby exacerbating the pathological progression of DPN (Graphic Abstract). Both in vitro cell experiments and in vivo animal studies have validated this mechanism. Silencing circ_0012856 significantly suppresses microglial M1 polarization and neuronal autophagy, alleviating the symptoms of DPN.

## AUTHOR CONTRIBUTIONS

Ji Chen, Yuanzhang Tang and Fengrui Yang conceived and designed the study. Fan Zhang, Yangyuxi Chen and Yingqing Lu performed the experiments. Xinxin Liu and Yuanzhang Tang analyzed the data. Ji Chen and Yuanzhang Tang wrote the manuscript. All authors reviewed and approved the final version of the manuscript.

## CONFLICT OF INTEREST STATEMENT

The authors declare no conflicts of interest.

## ETHICS STATEMENT

All animal experiments were approved by the Animal Ethics Committee of Hunan University of Medicine General Hospital (No. 202403055).

## Supporting information

Tables S1, S2

## Data Availability

All data can be provided as needed.
