# Supplementary material for: Role of circ_0012856 in modulating molecular pathways of diabetic peripheral neuropathy
Source: J Cell Commun Signal. 2025 Jun 12;19(2):e70019. doi: 10.1002/ccs3.70019 (PMC12162355; doi:10.1002/ccs3.70019)
Supplement: Supplementary file 1 — Tables S1, S2 [file CCS3-19-e70019-s001.docx]

**Table S1. RT-qPCR Primer Sequences.**

| **Gene** | **Sequence(5'-3')** |
| --- | --- |
| circ_0012856 (mouse) | Forward: CGGGTCATGTTTGCACAGTC |
|  | Reverse: AGCTAGCAGCAACTTCACGA |
| miR-124(mouse) | Forward: CGTGTTCACAGCGGACCTTGAT |
|  | Reverse: Universal Reverse Primer |
| EZH2 (mouse) | Forward: TCCTTCCATGCAACACCCAA |
|  | Reverse: GTGCTGGGTCTGCTACTGTT |
| STAT3 (mouse) | Forward: ACCAACGACCTGCAGCAATA |
|  | Reverse: TCCATGTCAAACGTGAGCGA |
| U6 (mouse) | Forward: ACCCCAGGATCACTCTACCC |
|  | Reverse: Universal Reverse Primer |
| GAPDH (mouse) | Forward: CCCTTAAGAGGGATGCTGCC |
|  | Reverse: TACGGCCAAATCCGTTCACA |

|  |
| --- |

**Table S2. Weight Changes of Mice in Different Groups**

| Group | Control | Model | Model + sh-NC  + NC antagomir | Model +  sh-circ_0012856 + NC | Model +  sh-circ_0012856 + miR-124 |
| --- | --- | --- | --- | --- | --- |
| Initial weight (g) | 20.5 ± 0.94 | 20.78 ± 0.9 | 20.6 ± 0.79 | 20.7 ± 1.04 | 20.93 ± 0.88 |
| 4-week weight (g) | 24.28 ± 1.58 | 18.03 ± 0.95 | 18.37 ± 0.79 | 20.12 ± 1.19 | 18.63 ± 0.8 |
| 8-week weight (g) | 26.77 ± 1.92 | 16.5 ± 0.92* | 16.87 ± 0.69* | 19.73 ± 1.06*# | 17.03 ± 0.8* |
| Blood glucose level (mM) | 5.26 ± 0.51 | 28.17 ± 2.79* | 27.53 ± 2.88* | 18.62 ± 1.65*# | 26.85 ± 2.46* |
| Weight change rate (%) | 31.56 | -20.22* | -18.28* | -4.6*# | -18.7* |

*Compared to Control, *P*<0.5; # Compared to the Model, *P*<0.5.
